# Grace Under Pressure: Resilience, Burnout, and Wellbeing in Frontline Workers in the United Kingdom and Republic of Ireland During the SARS-CoV-2 Pandemic

**DOI:** 10.3389/fpsyg.2020.576229

**Published:** 2021-01-27

**Authors:** Rachel C. Sumner, Elaine L. Kinsella

**Affiliations:** ^1^HERA Lab, School of Natural & Social Sciences, University of Gloucestershire, Cheltenham, United Kingdom; ^2^Department of Psychology, RISE Lab, Health Research Institute, Centre for Social Issues Research, University of Limerick, Limerick, Ireland

**Keywords:** coronavirus, Covid-19, CV19 heroes, heroism, keyworkers, government strategy, meaning in life

## Abstract

The coronavirus pandemic has necessitated extraordinary human resilience in order to preserve and prolong life and social order. Risks to health and even life are being confronted by workers in health and social care, as well as those in roles previously never defined as “frontline,” such as individuals working in community supply chain sectors. The strategy adopted by the United Kingdom (UK) government in facing the challenges of the pandemic was markedly different from other countries. The present study set out to examine what variables were associated with resilience, burnout, and wellbeing in all sectors of frontline workers, and whether or not these differed between the UK and Republic of Ireland (RoI). Individuals were eligible if they were a frontline worker (in health and social care, community supply chain, or other emergency services) in the UK or RoI during the pandemic. Part of a larger, longitudinal study, the participants completed an online survey to assess various aspects of their daily and working lives, along with their attitudes toward their government’s handling of the crisis, and measurement of psychological variables associated with heroism (altruism, meaning in life, and resilient coping). A total of 1,305 participants (*N* = 869, 66.6% from the UK) provided sufficient data for analysis. UK-based workers reported lower wellbeing than the RoI-based participants. In multivariate models, both psychological and pandemic-related variables were associated with levels of resilience, burnout, and wellbeing in these workers, but which pandemic-related variables were associated with outcomes differed depending on the country. The judgment of lower timeliness in their government’s response to the pandemic appeared to be a key driver of each outcome for the UK-based frontline workers. These findings provide initial evidence that the different strategies adopted by each country may be associated with the overall wellbeing of frontline workers, with higher detriment observed in the UK. The judgment of the relatively slow response of the UK government to instigate their pandemic measures appears to be associated with lower resilience, higher burnout, and lower wellbeing in frontline workers in the UK.

## Introduction

Keeping economies and societies afloat during crisis is a delicate balance between urging caution and responsibility, and deterring panic. In 2019, the first case of the Covid-19 disease (caused by the virus SARS-CoV-2) was diagnosed, and by 11th March 2020 the World Health Organisation (WHO) declared a global pandemic. Since then, most countries of the world have faced an unprecedented public health care crisis, where human behavior plays a critical role not only in the spread of disease, but also, in response to the crisis.

**FIGURE 1:**
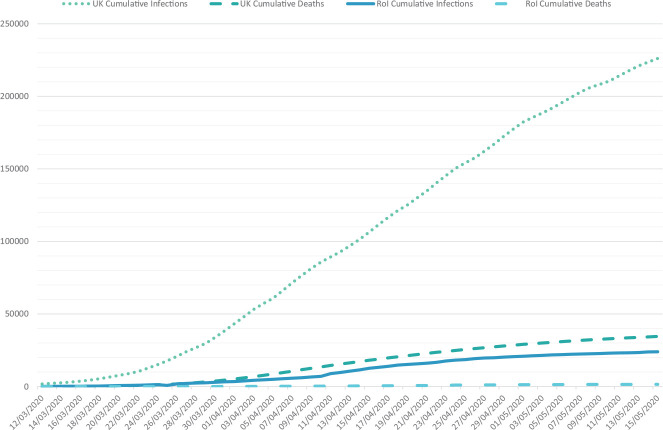
Cumulative number of infections and deaths in the United Kingdom (UK) (Data obtained from the United Kingdom Government Coronavirus surveillance data, available at: http://coronavirus.data.gov.uk/) and Republic of Ireland (Rol) (Data obtained from the Republic of Ireland Government Coronavirus surveillance data hub, available at: http://covid19ireland-geohive.hub.arcgis.com/) from COVID-19. Data obtained from NHS and HSE pandemic statistical reports.

After the WHO declared Covid-19 to be a pandemic on 11th March 2020, the leaders of many European governments addressed their countries to announce their strategies to take on the challenges of the pandemic. The leader of the United Kingdom (UK) government (Prime Minister Boris Johnson) advised that anyone with a new or persistent cough or fever should self-isolate; on this day, the approximate number of infections was 590, with a recorded eight deaths. The advice at this time was not to minimize gatherings of people, nor to close schools or businesses. On the same day, the leader (Taoiseach) of the Republic of Ireland (RoI) government, Dr. Leo Varadkar, announced the immediate closure of schools, colleges, and universities, and the limiting of public gatherings to those under 100 attendees in the case of indoor events, and under 500 in the case of outdoor events. At this point in RoI, the approximate number of infections was 70, with one recorded death. The UK government did not limit gatherings of any kind until an announcement on the evening of the 23rd March 2020, after which many large-scale sporting events were canceled by organizers, but others went ahead (such as The Cheltenham Festival, a 4-day horseracing event attended by approximately 251,684 individuals). The so-called “lockdown” measures — limiting individuals to working from home where possible, introducing furlough support to business, and limiting opportunities to leave the house for anything other than work or provisions to one outing for exercise only — were described by Prime Minister Johnson, to be a core component of the “delay” phase of the pandemic. These restrictions were placed in the UK on the evening of the 23rd March 2020, where the approximate number of infections were 6,650, with an approximate number of hospital fatalities at 335 (0.49/100,000). In contrast, similar measures were put in place in RoI on the 27th March 2020, when the approximate number of infections was 2,121 and the approximate number of fatalities was 22 (0.44/100,000). To provide a point of equal comparison, by the 22nd April 2020, the approximate morbidity rate in the UK was 133,495 to RoI’s 16,671, and the approximate mortality rate in the UK was 18,738 (27.61/100,000) to RoI’s 769 (15.57/100,000). See Figure 1 for an overview of the cumulative morbidity and mortality rate in the UK and RoI from the 12th March 2020 to 15th May 2020 derived from published government data. It is worth noting that on 5th May 2020, the death toll in the UK (29,427; 43.34/100,000) became the highest in Europe, and the second highest in the world at that point in time in the pandemic. Both countries have adopted markedly different public health strategies in relation to managing the outbreak of the disease, with the UK adopting an approach many have likened to a “herd immunity” strategy (Jetten et al., 2020), whereas the RoI adopted a more conservative approach more in line with WHO guidance. For our purpose, these strategic differences provide interesting comparative contexts for examining the psychological impact of working in a frontline capacity during the Covid-19 crisis.

During Covid-19, and other health crises, the term frontline workers (or frontline heroes) has been applied to workers that provide an essential service during the pandemic and lockdown periods across the world (Hsin and Macer, 2004; Smith et al., 2020). Frontline workers, health care workers in particular, have been likened in the media to combat veterans: minimizing their own distress in order to care for others, and hailed as heroes. The frontline workers in this global pandemic, predominantly in health and care settings, emergency services and community supply chain, have faced increased risks of contracting the virus themselves and spreading it to their significant others (Liu et al., 2020). They have also needed to navigate a range of exceptional challenges ranging from increased exposure to death in health and care home settings, increased hours and pressures at work, dealing with challenging situations brought on from contact with members of the public, and social isolation from colleagues and loved ones (Lai et al., 2020; Liu et al., 2020; Stuijfzand et al., 2020; The Lancet, 2020). The physical and psychological impact of working during the Covid-19 pandemic have been significant: globally, thousands of frontline workers have died from contracting SARS-CoV-2, and some have committed suicide (World Health Organization, 2020; Zaka et al., 2020). We know from previous research that there are significant mental health consequences associated with disasters (see Goldmann and Galea, 2014 for review), and for healthcare crises, the mental health fallout for healthcare professionals is likely to last beyond the physical threat of the virus itself (Maunder et al., 2006).

Existing research with frontline workers during health crises has been conducted almost exclusively with healthcare professionals. Experience with previous epidemics show that dealing with infected patients can cause considerable mental stress, high anxiety, and posttraumatic stress disorder (PTSD) for healthcare workers, especially nurses (Chersich et al., 2020; Tsamakis et al., 2020). Work carried out during the HIV/AIDS outbreak showed that anxiety, depression, and various personality factors associated with emotional processing and management of interpersonal relationships were some of the significant risk factors of highly “burned out” healthcare workers (Bellani et al., 1996). Many healthcare workers reported feelings of extreme vulnerability, uncertainty, psychological distress, and symptoms of anxiety during the outbreak of severe acute respiratory syndrome (SARS) (Tam et al., 2004). Most recently, research conducted during Covid-19 to-date indicates that those frontline healthcare workers are experiencing high rates of depression, distress, anxiety and insomnia (Lai et al., 2020). Longitudinal data from previous health crises indicate that the psychological impact of frontline healthcare workers is long-lasting, and that they are at increased risk of burnout, depression, anxiety, substance misuse, and PTSD over the longer term across epidemic surges and several years beyond (e.g., Ho et al., 2005; Wu et al., 2008).

It is perhaps not too surprising that negative psychological outcomes are common in frontline HCWs during both acute and post-acute phases of health crises. However, not all workers experience equal levels of distress, and some patterns of influencing factors have been identified. For example, the extent that healthcare workers perceive sufficiency of information during an influenza outbreak has been shown to relate to psychological distress (Goulia et al., 2010). In addition, workers’ appraisal of their own working conditions as high-risk relates to decreased levels of psychological resilience (Son et al., 2019). Some protective factors have been noted. For example, higher levels of social support have been associated with greater resilience and positive mental health in healthcare workers (Hou et al., 2020). Having an empathetic relationship with patients has been shown to reduce risk of burnout (Visintini et al., 1996). On the other hand, external coping style (e.g., religiosity, denial) has been shown to significantly predict levels of burnout beyond known factors such as age, perceived workload, and locus of control in caregivers (Gueritault-Chalvin et al., 2000). However, gaps in our understanding remain, such as the extent to which government policy may be associated with these outcomes. Also, it is not known whether others working on the frontline (beyond healthcare workers) are also vulnerable to these negative psychological outcomes, and what factors may be associated with their general welfare as they undertake this work. As a result, our knowledge of how best to support frontline workers across a range of essential service sectors is limited.

The Covid-19 pandemic is of an international scale not seen in other health crises in living memory, and the numbers of people working in frontline positions both in health and non-healthcare positions has been of a scale never witnessed before. As a result, there are likely to be additional factors that influence the mental health and wellbeing of frontline workers that have not been considered in previous research, such as their appraisal of their government’s response to the outbreak, and their uncertainty about whether they were infected with this extremely contagious (and sometimes asymptomatic) virus. The limited existing literature concerns the coping and wellbeing of healthcare workers during previous health crises but cannot account for the experiences of the additional sectors of frontline workers that the Covid-19 pandemic has brought about. Indeed, stressful working conditions have not been limited to healthcare settings — all types of frontline workers faced risks with regard to their health and the prospect of potentially infecting their loved ones. The consumer panic, for instance, at the prospect of needing to self-quarantine for several weeks put an enormous strain on workers in community supply chains (such as supermarket workers, delivery drivers, and postal workers). As well as better understanding how personal factors may be associated with the psychological response of workers across all frontline sectors, there is a pressing need to understand how wider contextual factors (such as government public health policies) play a part in these outcomes.

To address this gap, in the present research we aimed to understand how both personal factors and pandemic variables are associated with resilience, burnout and wellbeing in frontline workers in the UK and RoI. Specifically, given the difference in government strategy in tackling the pandemic between the two countries, we assessed participants’ perceptions of their government strategies (with respect to appropriateness, timeliness, and effectiveness) to further understand whether they may be related to the welfare of frontline workers. Further, given that uncertainty has been previously associated with resilience and burnout (Kimo Takayesu et al., 2014; Simpkin et al., 2018), and the fact that the beginning stages of the pandemic were characterized by a lack of available, accurate testing for SARS-CoV-2 infection, we were interested to see whether the uncertainty of having had the infection (which, at the time, was thought to be possible only once) may be associated with the stress of working on the frontline.

The personal variables of interest for the present study — meaning in life, altruism and resilient coping style — were selected in order to gain as much insight as possible into those factors that are associated with extremely stressful work, particularly work associated with heroic action. Meaning in life is the feeling that people have that their lives and experiences make sense and matter (Steger, 2009), which plays a role in human well-being (e.g., Zika and Chamberlain, 1992). Individuals differ in relation to how hard they search for meaning in their lives (Steger et al., 2006), and also, situational factors can trigger a search for meaning process (e.g., Van Tilburg and Igou, 2012; Maher et al., 2018). Search for meaning is associated with negative psychological states (Steger et al., 2006; Steger, 2009), unless presence of meaning is already high (Park et al., 2010). Behaving heroically may sometimes imbue life with meaning due to an increased sense of purpose and coherence, and at other times, decrease sense of meaning in life due to social ostracism and isolation from others (Kinsella et al., 2019). Interestingly, greater search for meaning is associated with greater motivation to behave heroically (Igou et al., 2018). Overall, the relationship between heroism and meaning in life is likely to be complicated: in the present study, meaning in life was included as a variable to further explore these relationships. Altruism was also included here in an exploratory capacity to see whether it may be associated with these outcomes, particularly burnout, as people who were more altruistic may be more likely to show higher levels of burnout due to going above and beyond the call of duty (e.g., working extra shifts, helping neighbors). The relationship between altruism and burnout appears to be quite complicated in the literature surrounding frontline work, with some citing it as protective and others as harmful (Altun, 2002; Burks and Kobus, 2012), so its incorporation in the present study came with no anticipations of directional relationship. Coping and resilience, whilst very much related, are distinct concepts. Coping is described to be an active and dynamic process of adjustment to challenge, whereas the concept of resilience has been defined as being the result of successful handling of challenge: encompassing recovery, recuperation, and regeneration following traumatic experiences (Earvolino-Ramirez, 2007; Hoge et al., 2007; Rice and Liu, 2016). Therefore, two distinct measures were included: a resilient coping measure was included to account for the use of coping strategies associated with delivering a status of resilience and a measure of resilience itself to incorporate a concept of invulnerability to these experiences of stress.

In addition to resilience, the other outcomes of interest were burnout and wellbeing. Burnout is associated with subjective wellbeing of those carrying out this vital work as well as work quality and workforce attrition (Maslach et al., 2001), so it is an important variable to consider within the context of the global pandemic – particularly when considering the associations with government strategy. When work demands surpass capacity, such as in the case of Covid-19, the conditions are ripe for burnout to occur. Wellbeing is a central aspect of the WHO definition of health, and is a core element of the WHO global strategy on occupational health for all (World Health Organization, 1995) and the World Health Assembly Worker’s Health strategy 2008–2017 (World Health Organization, 2007). The present study utilized wellbeing as a key outcome as assessing the wellbeing of frontline workers has been cited as an ethical duty both during and in the aftermath of Covid-19 (Gavin et al., 2020).

The present study was conducted during the earlier stages of the pandemic in Northern Europe (31st March to 15th May 2020) to understand the impact of working during the Covid-19 crisis on frontline workers. Here, we present the cross-sectional findings from the first registered study examining the mental health effects of working on the ‘frontline’ (including healthcare and non-healthcare workers) during the Covid-19 pandemic in the UK and RoI – two countries with markedly different public health strategies in response to the outbreak of Covid-19. In doing so, we respond to an urgent call for researchers to assess the psychological effects of Covid-19 on frontline workers (Holmes et al., 2020). This study is particularly novel in the sense that a broad spectrum of frontline workers were sampled, not limited to healthcare settings. Another novel aspect of this study is provided through a comparison of UK and RoI workers as we assessed how individuals rated their respective governmental strategies in dealing with the pandemic and their own certainty around Covid-19 diagnoses are associated with resilience, burnout and wellbeing in these workers.

## Materials and Methods

### Participants

Data collection commenced on 31st March 2020, 20 days after the WHO declared pandemic status for Covid-19, and 8 days after “lockdown” status was announced in the UK, and 4 days after a similar status was announced in RoI. Recruitment to the study concluded on 15th May 2020. Inclusion criteria were that participants were over 18 years old, working in a frontline role in the UK or RoI. Participants were advised of the nature of the study, that it would contain questions related to the pandemic, and were advised not to participate if they felt that they would be distressed as a result. The survey was presented online^1^.

### Measures

#### Participant Demographics

Demographic details were requested from participants in the form of age, gender, country of residence (UK or RoI), level of education, marital status, caring status (for children, relatives, or other adults), and employment sector (health and social care; supply chain; other emergency services; or other). For the purposes of defining these groups, examples of these groups were provided on the survey. For the Health and Social Care category these were: nurse, doctor, paramedic, care worker, pharmacist, allied health professional. For the supply chain group, the provided examples were: supermarket worker, food/grocery delivery driver, postal worker, convenience store workers, other food/grocery provision worker. The “other emergency services” group examples were: police, fire and rescue; and the final category of “other frontline key worker” invited participants to specify their role (this category included public transport operators, teachers, and veterinaries).

#### Pandemic-Related Variables

Information specific to the Covid-19 pandemic context was gathered. Participants were asked to rate on a scale of 1 (not at all) to 10 (very much so) whether they thought decisions made by their government and organization were: appropriate, timely, and effective. To understand whether social support might be associated with outcomes, participants were asked about their partnership status. To capture personal experience of Covid-19 infection, participants were asked if they, a family member, a friend, or a colleague had a Covid-19 infection (no; I’m not sure; yes – not tested but certain of diagnosis; yes – formally tested and diagnosed). Due to the lack of widespread testing for Covid-19, these measures were later collapsed to assess certainty (i.e., those indicating either “no” or one of the “yes” answers as certain, or “I’m not sure” being categorized as uncertain). As infection with SARS-CoV-2 may take some time before becoming symptomatic, if at all, it was important to assess this level of certainty around infection, as this would potentially have an impact on worry surrounding contracting or spreading the infection to others. It is also important to consider that certainty around infection can be addressed with sufficient availability of accurate testing, and so as a factor that may contribute to the outcomes of interest, it is also something that can provide learning from these early stages of the pandemic, and an important factor to consider if and when another similar emergency arises in the future. It is also possible that significant stress may be experienced just from having or not having had SARS-CoV-2, and so supplementary analyses were conducted treating this variable in an alternative means to understand incidence of infection (i.e., yes, no, I don’t know). These findings are presented in Supplementary Tables 1, 2.

#### Psychological Variables

To assess concepts associated with heroic and altruistic behaviors, the Meaning in Life Questionnaire (MLQ: Steger et al., 2006) and Adapted Self-Report Altruism Scale (ASRAS: Witt and Boleman, 2009) were used. The MLQ is a 10-item scale separated into two components: “presence,” an index of whether or not an individual feels they have found meaning in life; and “search,” whether the individual is still seeking meaning in life. The score ranges for each subdimension of the questionnaire are five to 35. Examples of items on the scale are: “My life has a clear sense of purpose” (presence) and “I am looking for something that makes my life feel meaningful” (search). Both subscales for the MLQ provided good internal consistency (α = 0.87; α = 0.90, respectively).

The ASRAS is a self-report measure of altruism, which although is different to heroism (Franco et al., 2011), examines the extent that have an ‘other-orientation’ and behave in ways that benefit others which is consistent with heroism. The ASRAS is a 14-item scale, with a score range of zero to 56, that asks participants to record the frequency of certain behaviors, for example: “I would donate clothes or goods to a charity,” and “I would help an acquaintance move houses.” This particular scale provided good internal consistency here (α = 0.88).

To assess coping styles associated with resilience the Brief Resilient Coping Scale (BRCS: Sinclair and Wallston, 2004) was used. The BRCS is a four-item scale (with observable range from four to 20) designed to assess individual tendencies to cope with stress in a highly adaptive manner, with items such as “I look for creative ways to alter difficult situation” and “I actively look for ways to replace the losses I encounter in life.” The originators (Sinclair and Wallston, 2004) suggest that levels of resilient coping can be conceptualized with reference to score ranges, in terms of those who are “low” (scoring between four and 13), “medium” (scoring between 14 and 16), and “high” (scoring from 17 to 20, inclusive). The scale captures specific patterns of stress adaptation that are more likely to result in increased resilience even in the face of highly stressful situations (Sinclair and Wallston, 2004). Here we use this scale as an indicator of individual differences in coping style that are associated with positive adjustment to life challenges. It was included as a separate variable due to its ability to be learned as a coping style (Polk, 1997) – this was important action as it provided a potentially useful avenue to explore and inform the development of future, evidence-based interventions to support frontline workers.

To assess the presence of resilience in participants, the Brief Resilience Scale (BRS: Smith et al., 2008) was included. Resilience, as measured by the BRS, is the present ability to recover from stress (Smith et al., 2008). Specifically, the BRS is designed to measure resilience — an individual’s ability to bounce back or recover from stress (Smith et al., 2008). The BRS is a six-item scale that asks respondents to indicate their agreement with statements such as: “It does not take me long to recover from a stressful event,” and “I usually come through difficult times with little trouble.” The BRS has a scale range of six to 30, but the final score is meaned as per author recommendations (Smith et al., 2008). Here, we use the BRS as both a predictor and outcome variable informed by research that shows that repeated engagement with stressors reinforce resilient traits and makes future resilience more likely (Woodgate, 1999). The BRCS and BRS scales were chosen due to their brevity in order to minimize participant burden and provided excellent reliability (α = 0.72; α = 0.86, respectively).

The main outcomes of interest for the present study were wellbeing and burnout, however, resilience (ability to bounce back or recover from stress; measured by the BRS: Smith et al., 2008) was also considered as an outcome. Wellbeing was measured by the Short Warwick Edinburgh Mental Wellbeing Scale (SWEMWBS: Tennant et al., 2007), and was chosen for its measurement of mental wellbeing that relates to both feelings and functioning, its brevity over the full version, and excellent internal consistency (α = 0.86). The SWEMWBS is a seven-item scale that asks participants to indicate their agreement to statements with regard to their experience in the preceding 2 weeks, with items such as “I’ve been feeling useful,” and “I’ve been dealing with problems well.” The scale has an observable range of seven to 35.

Burnout was measured by the Bergen Burnout Inventory (BBI: Salmela-Aro et al., 2011), chosen for its brevity but also for its sub-domains of exhaustion, cynicism, and feelings of inadequacy. The BBI is a nine-item scale, asking participants to indicate their agreement with statements in line with their experience in the last month, with items such as: “I feel dispirited at work and I think of leaving my job” (in the cynicism dimension), “I often sleep poorly because of the circumstances at work” (in the exhaustion dimension), and “My expectations to my job and to my performance have reduced” (in the inadequacy dimension). Total and mean scores were calculated for each of the subscales: exhaustion, cynicism, and feelings of inadequacy. Mean scores are presented for demographic overview for comparison to other samples, but total scores were used in multivariate analyses. Reliability analyses for these provided good metrics for the total scale (α = 0.86) as well as the subscales (exhaustion: α = 0.65; cynicism: α = 0.79; feelings of inadequacy: α = 0.72).

### Procedure

The present sample recruited frontline workers from the UK and RoI by opportunity and snowballing sampling through social and news media as part of a larger longitudinal study (the CV19 Heroes project^2^). For the purposes of the study, “frontline workers” were defined as “frontline health and social care workers; frontline workers in community supply chains: supermarket staff, delivery drivers, and stock management; and any other frontline workers during the pandemic such as police officers/Gardaí.” Participants were guided toward the online survey through Facebook, Twitter, Reddit, and news media advertising. The survey included a full participant information sheet, consent form, and debrief including adequate signposting for participants of both countries to access accurate information with regard to Covid-19 and psychosocial support in the case of any distress caused. The questionnaire itself was expected to take around 15 min for participants to complete. Any responses of potential participants that did not complete the survey in full were not recorded to allow participant withdrawal. The study was reviewed and ethically approved by the University of Gloucestershire School of Natural and Social Sciences Research Ethics Panel (NSS/2003/003), and protocol was registered on the Open Science Framework^3^ on March 23rd, 2020. To assist in reduction of potential study duplication, the study was also registered on a variety of Covid-19 research trackers.

### Analysis

All data were analyzed using SPSS version 23. Summary data regarding participant demographics, and tests of difference for comparison between the country of residence (UK or RoI) were carried out using one-way ANOVA or χ^2^ depending on the type of data in question.

To assess what psychological aspects may contribute to resilience, burnout, and wellbeing; regression models were fit including partnership status (as a proxy for social support), caring responsibilities (as a means of understanding additional stressors beyond working), meaning in life (presence and search), altruism, and resilient coping. In a nested approach, resilience was used as an additional predictor for burnout, and both resilience and burnout were added as predictors for wellbeing. To assess whether or not specific pandemic factors contributed to these outcomes, the pandemic associated factors (attitude toward government response measures, certainty of knowledge in self/family/friends/workers having been infected with SARS-CoV-2) were included in models to predict resilience, burnout, and wellbeing. To answer the research question concerning whether differential pandemic response strategy may contribute to these outcomes, models were then fit to include all predictors (person-specific and pandemic-specific) by country in a stratified approach.

## Results

### The Sample

A total of 1,318 individuals completed the online survey. During data cleaning, eight were removed that had not listed either the UK or RoI as their country of residence, and five participants were removed for not being classed as a frontline worker during the pandemic. A total of 1,305 participants remained within the dataset for analysis.

The majority of respondents were from the UK (*N* = 869, 66.6%), identified as female (*N* = 1109, 86.7%), identified as white (*N* = 1244, 95.3%), and reported being a frontline worker in the area of health and social care (*N* = 1039, 79.9%). The majority indicated that they had caring responsibilities alongside work (*N* = 789, 60.6%), the largest group within these were those with children (*N* = 439, 33.7%). The majority indicated that they had a partner either in marriage, civil partnership, or cohabitation (*N* = 861, 66.1%). Across the whole sample, and ranging from a score between 1 and 10, the participants rated their government’s response to the pandemic at 5.7 ± 3.33 for “appropriate,” 4.3 ± 3.16 for “timely,” and 5.1 ± 3.15 for “effective.” Participants were asked whether they, anyone in their family, their friends, or their colleagues had a Covid-19 infection, with the option of answering one of the following: “No,” “I’m not sure,” “Yes – not tested, but certain of diagnosis,” and “Yes – formally tested and diagnosed.” Due to the lack of availability of effective testing at the time of data collection, these categories were collapsed to operationalise certainty around diagnosis; with those indicating “no,” and either of the “yes” (i.e., “Yes – not tested, but certain of diagnosis,” and “Yes – formally tested and diagnosed”) options into a category of “certain,” and those selecting “I’m not sure” into a category of “uncertain.” For each category, certainty was the most populous, but this varied according to which individual was in question: self *N* = 908 (69.6%), family *N* = 1024 (78.6%), friends *N* = 993 (76.3%), and colleagues *N* = 972 (74.5%). Table 1 provides an overview of the sample. As previously noted, this concept of testing certainty may not be the only way to conceptualize stress in these frontline workers, and so multivariate analyses have been carried out using an operationalization that captures occurrence of Covid-19 infection and are presented in Supplementary Tables 1, 2.

**TABLE 1:** Demographic, psychological, and pandemic-factor overview of the sample, including United Kingdom (UK) and Republic of Ireland (Rol) subsamples and tests of difference between the subsamples.

		Whole Sample				UK				RoI				
		(*N* = 1305)				(*N* = 869, 66.6%)				(*N* = 436, 33.4%)				
		*N*	%	*M*	*SD*	*N*	%	*M*	*SD*	*N*	%	*M*	*SD*	Test of difference
Age				43.4	10.89			43.8	11.16			42.6	10.30	*F*_(1,1286)_ = 3.36, *p* = 0.067
Gender	Female	1109	86.7			733	86.2			376	87.6			χ^2^(5) = 2.37, *p* = 0.796
	Male	162	12.7			110	13.0			52	12.1			
	Trans woman	1	0.1			1	0.1							
	Trans man	1	0.1			1	0.1							
	Non-binary/Gender queer	2	0.2			2	0.2							
	Prefer not to say	4	0.3			3	0.4			1	0.2			
Employment division	Health and Social Care	1039	79.9			685	78.9			354	81.9			**χ^2^(3) = 10.67, *p* = 0.014**
	Supply chain	112	8.6			88	10.1			24	5.6			
	Other emergency services	59	4.5			33	3.8			26	6.0			
	Other frontline key worker	90	6.9			62	7.1			28	6.5			
Highest level of education	Primary	121	9.3			110	12.7			11	2.5			**χ^2^(5) = 75.18, *p* < 0.001**
	Secondary	284	21.9			219	25.3			65	14.9			
	Foundation degree/higher diploma	264	20.3			175	20.3			89	20.5			
	Undergraduate degree	386	29.7			230	26.6			156	35.9			
	Postgraduate degree	232	17.9			122	14.1			110	25.3			
	Doctoral degree	12	0.9			8	0.9			4	0.9			
Partnership status	Partnered	861	66.1			584	67.4			277	63.5			χ^2^(1) = 1.97, *p* = 0.172
	Unpartnered	441	33.9			282	32.6			159	36.5			
Caring responsibilities	Yes – children, parents, or other adults	789	60.6			499	57.6			290	66.7			**χ^2^(1) = 9.93, *p* = 0.001**
	No caring responsibilities	512	39.4			367	42.4			145	33.3			
Government response rating	Appropriate			5.7	3.33			5.2	3.19			6.8	3.35	***F*_(1,1303)_ = 70.23, *p* < 0.001**
	Timely			4.3	3.16			3.6	2.76			5.7	3.44	***F*_(1,1303)_ = 141.74, *p* < 0.001**
	Effective			5.1	3.15			4.6	2.98			6.0	3.25	***F*_(1,1303)_ = 66.38, *p* < 0.001**
Have you had CV19?	No	770	59.0			458	52.7			312	71.7			**χ^2^(3) = 50.93, *p* < 0.001**
	I’m not sure	396	30.4			307	35.3			89	20.5			
	Yes – not tested but certain	75	5.8			64	7.4			11	2.5			
	Yes – tested and diagnosed	63	4.8			40	4.6			23	5.3			
	Certain	908	69.6			562	64.7			346	79.5			**χ^2^(1) = 30.31, *p* < 0.001**
	Uncertain	396	30.4			307	35.3			89	20.5			
Has anyone in your family had CV19?	No	852	65.4			500	57.6			352	81.1			**χ^2^(3) = 75.51, *p* < 0.001**
	I’m not sure	278	21.4			228	26.3			50	11.5			
	Yes – not tested but certain	115	8.8			100	11.5			15	3.5			
	Yes – tested and diagnosed	57	4.4			40	4.6			17	3.9			
	Certain	1024	78.6			640	73.7			384	88.5			**χ^2^(1) = 37.47, *p* < 0.001**
	Uncertain	278	21.4			228	26.3			50	11.5			
Have any of your friends had CV19?	No	508	39.0			299	34.4			209	48.2			**χ^2^(3) = 58.05, *p* < 0.001**
	I’m not sure	309	23.7			237	27.3			72	16.6			
	Yes – not tested but certain	160	12.3			136	15.7			24	5.5			
	Yes – tested and diagnosed	325	25.0			196	22.6			129	29.7			
	Certain	993	76.3			631	72.7			362	83.4			**χ^2^(1) = 18.35, *p* < 0.001**
	Uncertain	309	23.7			237	27.3			72	16.6			
Have any of your colleagues had CV19?	No	314	24.1			168	19.3			146	33.6			**χ^2^(3) = 104.71, *p* < 0.001**
	I’m not sure	332	25.5			242	27.8			90	20.7			
	Yes – not tested but certain	194	14.9			182	20.9			12	2.8			
	Yes – tested and diagnosed	464	35.6			277	31.9			187	43.0			
	Certain	972	74.5			627	72.2			345	79.3			**χ^2^(1) = 7.83, *p* = 0.006**
	Uncertain	332	25.5			242	27.8			90	20.7			
Meaning in Life	Presence			26.4	6.40			26.0	6.52			27.3	6.05	***F*_(1, 1272)_ = 11.79, *p* = 0.001**
	Search			19.5	8.21			19.7	7.98			19.3	8.66	*F*_(1,1303)_ = 0.56, *p* = 0.454
Altruism				40.4	9.27			40.2	9.69			41.0	8.33	*F*_(1,1254)_ = 2.01, *p* = 0.156
Resilient Coping	Total			14.6	3.03			14.6	3.00			14.7	3.09	*F*_(1, 1303)_ = 0.79, *p* = 0.373
	Low resilient coper	400	30.8			275	31.7			125	28.9			χ^2^(2) = 1.26, *p* = 0.532
	Medium resilient coper	569	43.8			377	43.5			192	44.3			
	High resilient coper	331	25.5			215	24.8			116	26.8			
Resilience				3.3	0.82			3.2	0.82			3.3	0.81	*F*_(1,1282)_ = 1.97, *p* = 0.161
Burnout	Total (mean)			3.1	1.10			3.2	1.06			3.1	1.18	*F*_(1,1303)_ = 1.94, *p* = 0.164
	Exhaustion			3.4	1.22			3.5	1.19			3.4	1.26	*F*_(1,1303)_ = 2.02, *p* = 0.156
	Cynicism			2.9	1.35			2.9	1.33			2.8	1.39	*F*_(1,1303)_ = 3.12, *p* = 0.078
	Feelings of inadequacy			3.1	1.27			3.1	1.24			3.1	1.34	*F*_(1,1303)_ = 0.15, *p* = 0.698
Wellbeing	SWEMWBS* Total			22.7	4.91			22.3	4.94			23.8	4.69	***F*_(1,1293)_ = 26.92, *p* < 0.001**
	SWEMWBS metric score			21.0	3.89			20.6	3.91			21.8	3.74	***F*_(1, 1293)_ = 24.93, *p* < 0.001**

Tests of difference marked in bold denote a significant difference (p < 0.05). *SWEMWBS, Short Warwick Edinburgh Mental Wellbeing Scale.

To examine basic associations with outcomes, a series of two-tailed zero-order correlations were implemented (see Table 2). Here, the personal factors of presence of and search for meaning in life, altruism, and resilient coping were associated with resilience and wellbeing to varying degrees from small to large effects (Cohen, 1988). The pandemic factors were associated with all outcomes, aside from the judgment of the appropriateness of their government’s response and resilience. The remaining relationships were significant, but with small effect sizes. For burnout, the only two personal factors that were significantly related were the two aspects of meaning in life, with *presence of meaning* being negatively related and *search for meaning* being positively related.

**TABLE 2:** Zero-order correlations of personal factors and resilience, burnout, and wellbeing in the whole frontline worker sample.

		Resilience (BRS)	Burnout (BBI, Total)	Wellbeing (SWEMWBS)
Presence of meaning in life (MLQ P)		0.348***	−0.291***	0.465***
Search for meaning in life (MLQ S)		−0.203***	0.264***	−0.195***
Altruism (ASRAS)		0.094**	–0.025	0.144***
Resilient coping (BRCS)		0.409***	–0.011	0.339***
Government response rating	Appropriate	0.044	−0.086**	0.152***
	Timely	0.060*	−0.114***	0.197***
	Effective	0.066*	−0.093**	0.182***
*Significant at *p* < 0.05				
**Significant at *p* < 0.01				
***Significant at *p* < 0.001				

*MLQ, Meaning in Life Questionnaire;ASRAS, Adapted Self-Report Altruism Scale; BBI, Bergen Burnout Inventory; BRCS, Brief Resilient Coping Scale; BRS, Brief Resilience Scale; P, presence; S, search; SWEMWBS, Short Warwick Edinburgh Mental Wellbeing Scale*.

### Comparisons Between the UK and the Republic of Ireland

In terms of the sample, there were significance differences in employment division [χ^2^(3) = 10.67, *p* = 0.014], with slightly more healthcare workers proportionately in the frontline sample of workers from RoI, and comparatively fewer from other groups. Education also differed between the two groups of participants [χ^2^(5) = 75.18, *p* < 0.001] with lower levels of education more frequently reported in the UK-based sample. Caring responsibilities between the two countries differed [χ^2^(1) = 9.93, *p* = 0.001], with notably higher levels of UK-based respondents indicating that they currently did not have caring responsibilities.

Differences were reported in each of the measures concerning the respondents’ rating of their government’s response to the Covid-19 crisis. Here, UK-based participants reported their government’s response to be significantly less appropriate [*F*_(1,1303)_ = 70.23, *p* < 0.001], timely [*F*_(1,1303)_ = 141.74, *p* < 0.001], and effective [*F*_(1,1303)_ = 66.38, *p* < 0.001] than did the RoI-based frontline worker sample. There were differences across the board between the countries for whether or not participants had either themselves [χ^2^(3) = 50.93, *p* < 0.001], their family members [χ^2^(3) = 75.51, *p* < 0.001], friends [χ^2^(3) = 58.05, *p* < 0.001], or colleagues [χ^2^(3) = 104.71, *p* < 0.001] contracted Covid-19. For each person considered, the certainty in whether or not they had experienced an infection was significantly greater in the RoI-based sample {self: [χ^2^(1) = 30.31, *p* < 0.001]; family: [χ^2^(1) = 37.47, *p* < 0.001]; friends [χ^2^(1) = 18.35, *p* < 0.001]; colleagues [χ^2^(1) = 7.83, *p* = 0.006]}.

For the psychological variables, only presence of meaning in life and wellbeing showed significant differences, with respondents from the UK reporting lower levels of presence of meaning in life [*F*_(1,1272)_ = 11.793, *p* = 0.001], and wellbeing in both raw SWEMWBS scores [*F*_(1,1293)_ = 26.92, *p* < 0.001] and their metric equivalents [*F*_(1,1293)_ = 24.93, *p* < 0.001]. Compared to population norm values (reported as 23.6 ± 3.90: Craig et al., 2011), the whole sample scored lower, but the RoI-based subsample scored comparatively close. Compared to other population samples of burnout using the BBI, the present sample scored higher on the total mean score (cited as 2.56 in workers from “social affairs and health”) and the mean scores for each of the burnout subscales (Exhaustion: 2.79, Cynicism: 2.26, Inadequacy: 2.66; Maarit et al., 2013), although this did not differ by country. Similarly, resilience was lower amongst the present sample than in other population norms (cited as 3.35: Kunzler et al., 2018), although this did not vary significantly between the two subgroups.

### Factors Associated With Resilience, Burnout, and Wellbeing

#### Resilience

For resilience, the model was significant [*F*_(13,1207)_ = 33.31, *p* < 0.001, *R*^2^ = 0.26, *R*^2^_*adjusted*_ = 0.26], with both presence of meaning in life (β = 0.02, *t* = 6.68, *p* < 0.001) and resilient coping (β = 0.11, *t* = 13.97, *p* < 0.001) positively associated with resilience. Search for meaning in life (β = −0.02, *t* = −6.69, *p* < 0.001) and SARS-CoV-2 infection certainty for self (β = −0.11, *t* = −2.07, *p* = 0.039) were negatively associated with resilience. In supplementary analyses, where the SARS-CoV-2 infection was treated with an operationalization that captured presence of virus rather than certainty (i.e., “Yes – not tested, but certain of diagnosis,” and “Yes – formally tested and diagnosed” were collapsed into a “yes” category, and both “no” and “I don’t know” remained, with “no” forming the reference group), the model for resilience showed the same variables as being associated with the outcome, in the same direction and to similar effect. Here, whether or not they themselves had had SARS-CoV-2 infection was also negatively associated with resilience (β = −0.08, *t* = −2.29, *p* = 0.022). Supplementary analyses for the whole sample are presented in Supplementary Table 1.

#### Burnout

Burnout was significantly predicted by the personal and pandemic-related factors [*F*_(__14,1206__)_ = 19.33, *p* < 0.001, *R*^2^ = 0.18, *R*^2^_*adjusted*_ = 0.17], with being partnered (β = 1.63, *t* = 2.89, *p* = 0.004), having higher levels of search for meaning in life (β = 0.15, *t* = 4.32, *p* < 0.001), and SARS-CoV-2 infection certainty for self (β = 2.04, *t* = 3.13, *p* = 0.002) being associated with higher total burnout. Having both higher presence of meaning in life (β = −0.30, *t* = −6.57, *p* < 0.001), resilience (β = −2.73, *t* = −7.58, *p* < 0.001), and perception of the timeliness of government actions (β = −0.40, *t* = −2.82, *p* = 0.002) were negatively associated with burnout. In supplementary analyses, the picture for burnout appears to change somewhat with regard to the variables that capture SARS-CoV-2 infection. Here, we also see that having had SARS-CoV-2 themselves (β = 1.15, *t* = 2.68, *p* = 0.008), or their friends (β = 0.75, *t* = 2.39, *p* = 0.017), or colleagues (β = 0.80, *t* = 2.34, *p* = 0.019) having had the infection also appeared to be associated with burnout.

#### Wellbeing

Personal and pandemic-related factors significantly predicted outcome wellbeing in the total sample of this study [*F*_(__15,1205__)_ = 85.28, *p* < 0.001, *R*^2^ = 0.52, *R*^2^_*adjusted*_ = 0.51]. Here, presence of meaning in life (β = 0.15, *t* = 8.01, *p* < 0.001), resilient coping (β = 0.27, *t* = 6.43, *p* < 0.001), resilience (β = 1.56, *t* = 10.95, *p* < 0.001), and perception of the timeliness of government actions (β = 0.16, *t* = 2.84, *p* = 0.005) were positively associated. Level of burnout (β = −0.19, *t* = −16.67, *p* < 0.001) was the only variable negatively associated with wellbeing.

Table 3 details the regression models for resilience (1), burnout (2), and wellbeing (3).

**TABLE 3:** Regression models examining separate contributions of personal and pandemic factors for resilience, burnout, and wellbeing in the whole frontline worker sample.

		Model 1					Model 2					Model 3				
		Resilience					Burnout^†^					Wellbeing				
		*F*_(13,1207)_ = 33.31, *p* < 0.001,					*F*_(14,1206)_ = 19.33, *p* < 0.001,					*F*_(15,1205)_ = 85.28, *p* < 0.001,				
		*R*^2^ = 0.26, *R*^2^_*adj*_ = 0.26					*R*^2^ = 0.18, *R*^2^_*adj*_ = 0.17					*R*^2^ = 0.52, *R*^2^_*adj*_ = 0.51				
		β	*t*	*p*	95% CI		β	*t*	*p*	95% CI		β	*t*	*p*	95% CI	
					Lower	Upper				Lower	Upper				Lower	Upper
	Partnership status	0.003	0.057	0.955	–0.086	0.091	1.634	2.890	**0.004**	0.525	2.742	0.195	0.887	0.375	–0.237	0.627
	Caring status	–0.011	–0.265	0.791	–0.097	0.074	0.840	1.551	0.121	–0.222	1.903	–0.160	–0.761	0.447	–0.573	0.253
	MLQ presence	0.024	6.676	**<0.001**	0.017	0.031	–0.303	–6.570	**<0.001**	–0.393	–0.212	0.146	8.012	**<0.001**	0.110	0.181
	MLQ search	–0.019	–6.686	**<0.001**	–0.024	–0.013	0.153	4.318	**<0.001**	0.084	0.223	–0.008	–0.606	0.545	–0.036	0.019
	Altruism	–0.003	–1.234	0.218	–0.007	0.002	0.006	0.193	0.847	–0.051	0.063	0.020	1.735	0.083	–0.003	0.042
	Resilient coping	0.111	13.973	**<0.001**	0.095	0.127	0.145	1.351	0.177	–0.065	0.354	0.267	6.431	**<0.001**	0.186	0.349
	Resilience*						–2.725	–7.575	**<0.001**	–3.431	–2.020	1.564	10.947	**<0.001**	1.284	1.844
	Burnout**											–0.186	–16.658	**<0.001**	–0.208	–0.164
Government response rating	Appropriate	–0.009	–0.782	0.435	–0.031	0.013	0.017	0.123	0.902	–0.0260	0.294	–0.036	–0.658	0.510	–0.143	0.071
	Timely	0.014	1.234	0.217	–0.008	0.037	–0.403	–2.816	**0.005**	–0.683	–0.122	0.158	2.835	**0.005**	0.049	0.267
	Effective	0.007	0.551	0.582	–0.018	0.032	0.069	0.436	0.663	0.243	0.382	0.045	0.731	0.465	–0.076	0.166
CV19 Infection certainty	Self	–0.107	–2.067	**0.039**	–0.209	–0.005	2.036	3.134	**0.002**	0.762	3.311	0.417	1.648	0.100	–0.079	0.914
	Family	0.013	0.227	0.820	–0.101	0.127	–0.201	–0.276	0.782	–1.626	1.224	–0.314	–1.114	0.266	–0.867	0.290
	Friends	0.005	0.098	0.922	–0.097	0.107	–0.718	–1.102	0.271	–1.996	0.560	–0.399	–1.579	0.115	–0.896	0.097
	Co-Workers	0.026	0.528	0.598	–0.071	0.123	–0.133	–0.216	0.829	–1.343	1.076	–0.194	–0.812	0.417	–0.664	0.275
	*Models 2 and 3 only															
	**Model 3 only															

*Significant differences are highlighted in bold.*^†^Burnout models were fit using the total (unmeaned) Bergen Burnout Inventory score. *MLQ, Meaning in Life Questionnaire. Resilient coping refers to specific adaptational styles associated with coping that are supportive of resilience. Resilience refers to the status of having successfully handled stressful situations*.

### Comparing Profiles of Association With Resilience, Burnout, and Wellbeing Between Those in the UK and Those in RoI

#### Resilience

Both models for resilience in the UK-based [*F*_(13,809)_ = 23.35, *p* < 0.001, *R*^2^ = 0.27, *R*^2^_*adjusted*_ = 0.26] and RoI-based [*F*_(13,384)_ = 11.26, *p* < 0.001, *R*^2^ = 0.28, *R*^2^_*adjusted*_ = 0.25] frontline workers were significant. For the UK workers, resilience was positively associated with presence of meaning in life (β = 0.02, *t* = 5.06, *p* < 0.001), higher levels of resilient coping (β = 0.11, *t* = 12.01, *p* < 0.001), and judgments of the government’s response as being more timely (β = 0.03, *t* = 1.99, *p* = 0.047). Search for meaning in life was negatively associated with resilience (β = −0.02, *t* = −6.11, *p* < 0.001) in this group. For the RoI-based frontline workers, the personal factors of presence of meaning in life (β = 0.03, *t* = 4.01, *p* < 0.001) and resilient coping (β = 0.11, *t* = 6.89, *p* < 0.001) were positively associated with resilience. In this subgroup, the pandemic-related factors of certainty over the experience of SARS-CoV-2 infection in self and family were differentially associated with resilience. Here, uncertainty of infection for self was negatively associated (β = −0.29, *t* = −2.90, *p* = 0.004), but uncertainty of infection in a family member was positively associated (β = 0.34, *t* = 2.61, *p* = 0.009) with wellbeing.

#### Burnout

Burnout was significantly predicted by the combined models for both UK-based [*F*_(14,808)_ = 11.70, *p* < 0.001, *R*^2^ = 0.17, *R*^2^_*adjusted*_ = 0.15] and RoI-based [*F*_(14,383)_ = 9.19, *p* < 0.001, *R*^2^ = 0.25, *R*^2^_*adjusted*_ = 0.22] frontline workers. For both groups of frontline workers, the pattern of significant personal factors predicting burnout was largely the same, with burnout being higher in those who were partnered (UK: β = 1.53, *t* = 2.27, *p* = 0.023; RoI: β = 2.09, *t* = 2.00, *p* = 0.046) and those whose search for meaning in life was higher (UK: β = 0.17, *t* = 4.02, *p* < 0.001; RoI: β = 0.13, *t* = 2.01, *p* = 0.046); and lower in those scoring more highly on presence of meaning in life (UK: β = −0.27, *t* = −5.05, *p* < 0.001; RoI: β = −0.37, *t* = −4.09, *p* < 0.001) and on resilience (UK: β = −2.35, *t* = −5.43, *p* < 0.001; RoI: β = −3.32, *t* = −5.08, *p* < 0.001). In a slight difference between the two groups, resilient coping styles were associated with higher levels of burnout in UK-based participants only (β = 0.25, *t* = 2.01, *p* = 0.045). For the pandemic associated factors, the UK-based participants reported higher levels of burnout if they judged the timeliness of their government’s response to the pandemic to be lower (β = −0.55, *t* = −2.86, *p* = 0.004), and if there was more uncertainty over whether they had themselves experienced Covid-19 (β = 1.49, *t* = 1.98, *p* = 0.048). For the frontline workers based in RoI, the only pandemic-related factor that predicted their levels of burnout in the model was uncertainty over whether they had themselves been infected with SARS-CoV-2 (β = 3.52, *t* = 2.68, *p* = 0.008).

#### Wellbeing

Both the UK-based [*F*_(15,807)_ = 55.32, *p* < 0.001, *R*^2^ = 0.51, *R*^2^_*adjusted*_ = 0.50] and RoI-based [*F*_(15,382)_ = 28.27, *p* < 0.001, *R*^2^ = 0.53, *R*^2^_*adjusted*_ = 0.51] frontline workers’ wellbeing was significantly predicted by the composite models of personal and pandemic factors. For both samples of frontline workers, presence of meaning in life (UK: β = 0.16, *t* = 7.30, *p* < 0.001; RoI: β = 0.10, *t* = 2.90, *p* = 0.004), resilient coping (UK: β = 0.24, *t* = 4.87, *p* < 0.001; RoI: β = 0.32, *t* = 4.17, *p* < 0.001), and resilience (UK: β = 1.64, *t* = 9.34, *p* < 0.001; RoI: β = 1.36, *t* = 5.47, *p* < 0.001) were positively associated, and burnout (UK: β = −0.19, *t* = −13.19, *p* < 0.001; RoI: β = −0.20, *t* = −10.57, *p* < 0.001) was negatively associated with wellbeing. For the UK-based sample, the perception of timeliness of the government’s response to the pandemic was positively associated with wellbeing (β = 0.16, *t* = 2.03, *p* = 0.043). There were no pandemic-associated factors associated with wellbeing in the model for the RoI-based frontline workers.

Table 4 details the regression models for resilience (4), burnout (5), and wellbeing (6), stratified by country (a: UK; b: RoI). Supplementary analyses for the stratified models can be found in Supplementary Table 2.

**TABLE 4:** Stratified regression models to examine the combined associations of personal and pandemic factors for the United Kingdom (UK)-based **(A)** and Republic of Ireland (RoI)-based **(B)** subsamples for resilience (4), burnout (5), and wellbeing (6).

		Resilience					Burnout^†^					Wellbeing				
		Model 4a – UK					Model 5a – UK					Model 6a – UK				
		*F*_(13,809)_ = 23.35, *p* < 0.001,					*F*_(14,808)_ = 11.70, *p* < 0.001,					*F*_(15,807)_ = 55.32, *p* < 0.001,				
		*R*^2^ = 0.27, *R*^2^_*adj*_ = 0.26					*R*^2^ = 0.17, *R*^2^_*adj*_ = 0.15					*R*^2^ = 0.51, *R*^2^_*adj*_ = 0.50				
		β	*t*	*p*	95% CI		β	*t*	*p*	95% CI		β	*t*	*p*	95% CI	
					Lower	Upper				Lower	Upper				Lower	Upper
	Partnership status	–0.005	–0.093	0.926	–0.113	0.102	1.534	2.273	**0.023**	0.210	2.859	0.075	0.279	0.781	–0.454	0.605
	Caring binary	0.025	0.486	0.627	–0.077	0.128	0.699	1.087	0.278	–0.563	1.960	–0.229	–0.895	0.371	–0.733	0.274
	MLQ presence	0.022	5.064	**<0.001**	0.013	0.030	–0.273	–5.053	**<0.001**	–0.378	–0.167	0.159	7.299	**<0.001**	0.116	0.202
	MLQ search	–0.021	–6.108	**<0.001**	–0.027	–0.014	0.172	4.019	**<0.001**	0.088	0.256	–0.011	–0.612	0.541	–0.044	0.023
	Altruism	–0.004	–1.505	0.133	–0.009	0.001	–0.020	–0.589	0.556	–0.086	0.046	0.020	1.520	0.129	–0.006	0.047
	Resilient Coping	0.112	12.011	**<0.001**	0.094	0.131	0.251	2.010	**0.045**	0.006	0.497	0.243	4.866	**<0.001**	0.145	0.341
	Resilience*						–2.353	–5.430	**<0.001**	–3.203	–1.502	1.641	9.337	**<0.001**	1.296	1.986
	Burnout**											–0.185	–13.194	**<0.001**	–0.212	–0.157
Government response rating	Appropriate	–0.017	–1.156	0.248	–0.046	0.012	0.108	0.600	0.548	–0.245	0.462	–0.053	–0.742	0.458	–0.194	0.088
	Timely	0.031	1.991	**0.047**	0.000	0.061	–0.545	–2.858	**0.004**	–0.919	–0.171	0.155	2.031	**0.043**	0.005	0.305
	Effective	0.010	0.608	0.543	–0.022	0.041	–0.004	–0.022	0.982	–0.393	0.384	0.062	0.787	0.431	–0.093	0.217
CV19 Infection certainty	Self	–0.049	–0.804	0.421	–0.169	0.071	1.494	1.982	**0.048**	0.014	2.973	0.419	1.392	0.164	–0.172	1.010
	Family	–0.075	–1.141	0.254	–0.204	0.054	0.315	0.697	0.697	–1.274	1.904	–0.183	–0.569	0.570	–0.817	0.450
	Friends	–0.018	–0.301	0.764	–0.137	0.101	–0.668	–0.893	0.372	–2.136	0.800	–0.576	–1.933	0.054	–1.161	0.009
	Co-Workers	0.026	0.441	0.659	–0.089	0.141	0.393	0.544	0.587	–1.026	1.813	0.055	0.190	0.849	–0.511	0.621
	Partnership status	0.004	0.049	0.961	–0.156	0.164	2.088	2.002	**0.046**	0.037	4.139	0.645	1.672	0.095	–0.114	1.404
	Caring binary	–0.119	–1.491	0.137	–0.275	0.038	0.869	0.850	0.396	–1.142	2.881	–0.291	–0.772	0.441	–1.032	0.450
	MLQ presence	0.027	4.009	**<0.001**	0.014	0.041	–0.366	–4.090	**<0.001**	–0.542	–0.190	0.097	2.895	**0.004**	0.031	0.164
	MLQ search	–0.015	–3.093	0.002	–0.025	–0.006	0.130	2.006	**0.046**	0.003	0.257	–0.006	–0.200	0.795	–0.053	0.041
	Altruism	0.002	0.534	0.594	–0.006	0.011	0.072	1.262	0.208	–0.040	0.185	0.018	0.865	0.387	–0.023	0.060
	Resilient Coping	0.105	6.885	**<0.001**	0.075	0.135	–0.198	–0.957	0.339	–0.604	0.209	0.318	4.171	**<0.001**	0.168	0.468
	Resilience*						–3.324	–5.078	**<0.001**	–4.611	–2.037	1.361	5.467	**<0.001**	0.871	1.850
	Burnout**											–0.199	–10.572	**<0.001**	–0.236	–0.162
Government response rating	Appropriate	0.002	0.099	0.921	–0.034	0.037	–0.162	–0.695	0.487	–0.619	0.296	–0.051	–0.589	0.556	–0.219	0.118
	Timely	–0.006	–0.324	0.746	–0.043	0.047	–0.408	–1.710	0.088	–0.877	0.061	0.086	0.971	0.332	–0.088	0.259
	Effective	0.005	0.237	0.813	–0.037	0.047	0.227	0.838	0.403	–0.306	0.761	0.049	0.492	0.623	–0.147	–0.246
CV19 Infection certainty	Self	–0.293	–2.902	**0.004**	–0.492	–0.095	3.516	2.683	**0.008**	0.940	6.093	0.494	1.014	0.311	–0.463	1.451
	Family	0.341	2.612	**0.009**	0.084	0.597	–0.163	–0.097	0.923	–3.482	3.155	–0.246	–0.396	0.692	–1.467	0.976
	Friends	0.043	0.421	0.674	–0.159	0.245	–0.716	–0.543	0.588	–3.310	1.878	0.203	0.418	0.676	–0.752	1.158
	Co-Workers	–0.002	–0.024	0.981	–0.182	0.178	–1.144	–0.973	0.331	–3.457	1.168	–0.812	–1.874	0.062	–1.664	0.040
*Models 5 and 6 only																
**Model 6 only																

*Significant differences are highlighted in bold.*

*^†^Burnout models were fit using the total (unmeaned) Bergen Burnout Inventory score.*

*MLQ, Meaning in Life Questionnaire.*

*Resilient coping refers to specific adaptational styles associated with coping that are supportive of resilience. Resilience refers to the status of having successfully handled stressful situations*.

## Discussion

From the onset of the Covid-19 global pandemic, frontline workers have been asked to work in conditions that put them at risk both physically and psychologically (see Kröger, 2020). As part of a larger project, the present study sought to understand those factors that were associated with resilience, burnout, and wellbeing in frontline workers in the UK and RoI, and whether they varied by country.

Overall, both samples of frontline workers had comparable levels of resilience and burnout, but the UK-based workers appeared to have significantly lower wellbeing. RoI-based workers were more likely to also be in an informal caring role, although this did not appear to be associated with any of the outcomes in the models, which is inconsistent with previous related findings (May et al., 2004). In terms of factors associated with the pandemic, UK-based workers reported lower levels of appropriateness, timeliness, and effectiveness of their government’s response to the pandemic than did those in RoI. UK-based workers were also more likely to be uncertain as to whether they, their family members, friends, or colleagues had experienced Covid-19. There were few differences in the regression models between the countries with reference to personal factors. The judgment of lower timeliness in their government’s response appeared to be an important factor for UK-based frontline workers. It was a significant predictor of resilience, burnout, and wellbeing in cumulative models, appearing to drive the overall association with wellbeing both independently, and as a function of its contribution to lower resilience and higher burnout. The RoI-based subsample were largely normative in their overall wellbeing, and this appeared to be borne out in cumulative models as there were no pandemic-associated factors (the only other bivariate differences between the countries) that were significant in the final model. The uncertainty of whether or not they themselves had experienced Covid-19 appeared to be a key driver for resilience, and for its cumulative contribution to burnout, but its associated with wellbeing was eradicated in the last model, where personal factors appeared to carry the total associative weight. For the workers in RoI, certainty over family members having had Covid-19 was positively associated with resilience, suggesting that the availability of reliable testing (for self or family members) may be an important aspect of resilience beyond personal factors.

The present findings both support and extend similar work in the field. We have observed lower resilience, higher burnout, and lower wellbeing in this sample of frontline workers in the UK and RoI during the Covid-19 pandemic. This aligns with prior work observing similar outcomes in healthcare workers (Tam et al., 2004; Chan et al., 2005; Maunder et al., 2006; Lung et al., 2009; Chersich et al., 2020) and extends this to broader sectors of frontline workers in this new global infectious disease pandemic^4^. The integration of personal factors along with pandemic-related factors provides the present work with findings that are meaningful for policy and practice. The examination of differences between samples from two countries whose strategies to delay, but work toward herd immunity (UK) or delay and eliminate (RoI) the virus have provided a unique opportunity to explore whether these differences are manifest in the psychological profiles of frontline workers. Both countries are, arguably, culturally and economically similar, providing a relatively stable basis for comparison.

The present examination of participant’s assessment of the government response to the pandemic provides the literature with a first glimpse at how government strategy might impact the health and wellbeing of those staffing its frontline; from the healthcare workers that tend to the infected, to the supermarket workers confronted with panic-buying and hoarding, to the workers who have stepped forward to provide auxiliary services in a time of need. Here, we observe differential patterns of variable association with each outcome by country, most particularly with regard to the pandemic factors, which may reflect some of the differences in the way that the pandemic has progressed in each country. In the RoI-based subsample, pandemic factors associated with judgment of the government strategy were not ultimately implicated in the outcomes in each model, however, uncertainty regarding experience of SARS-CoV-2 infection were significantly associated with both resilience and wellbeing. This could be explained by related literature that has explored the impact of fearing passing the infection on to others (Tam et al., 2004; Maunder et al., 2006), and could also explain why being partnered appears to be positively associated with burnout in the whole sample and in each country-based subsample.

Compared to related literature examining the factors associated with heroic action, the present findings appear to both complement and refute previously observed trends. The lack of relative importance for altruism as a factor in determining variance in resilience and wellbeing in these workers appears to contravene previous studies. In related work, Vaulerin et al. (2016) determined that burnout was not sufficiently predicted by the personality facet of agreeableness, of which altruism is one component. Whilst altruism has been shown to be protective of health and wellbeing, its salutogenic impact may well be over-ridden when the task at hand (particularly one of helping or assistance) proves to be overwhelming (Post, 2005). In this context, it would seem with our present population that altruism in the face of the significant adversity faced may not be protective, particularly over the longer term. With regard to meaning in life, the present findings echo others in the field. Presence of meaning in life has been suggested to be protective against burnout in palliative care nurses (Gama et al., 2014) and firefighters (Krok, 2016). Meaning in life is a relatively dynamic concept within the sphere of work. Engaging in activities perceived to be meaningful has been noted to have longitudinal correlates of presence of meaning (Eakman, 2014), and for some employment sectors such as healthcare, the opportunity to engage in a work that answers a “calling” not only provides meaning, but may also protect from burnout (Vinje and Mittelmark, 2007). However, encountering particularly challenging circumstances can damage meaning, and result in losing a feeling of having meaning in life, and therefore necessitate an increase in search for meaning (Hicks and King, 2009). For our present sample, it is likely that engaging in meaningful and valuable work for the current context may have increased presence of meaning in life for some, but also that challenges associated with the pandemic (such as the witnessing of death, the experience of customer hoarding, or experiencing the use of coughing or spitting as a means of social protest) may also impair meaning. Here, we find that both search for and presence of meaning in life are differentially associated with all outcomes in the whole sample, and when stratified by sample location. In other words, those experiencing high levels of search for meaning in life appear to have poorer resilience, burnout, and wellbeing, and those higher in presence the inverse.

Our use of cumulative models of wellbeing (where resilience contributes to burnout, and both resilience and burnout contribute to wellbeing) provide a new perspective for understanding the mental health of frontline workers, as well as providing greater clarity about the relationships between these psychological constructs more broadly. Here, we are able to determine the cumulative contribution of personal factors and pandemic-related factors on wellbeing. The examination of resilient coping style as distinct from the concept of resilience provides new knowledge to the field, in terms of being able to provide intervention avenues for those working on the frontline. The personal factors that are associated with each of the outcomes (presence of and search for meaning in life, and resilient coping style) are associated with each outcome in each country-based subsample. The addition of pandemic-associated factors to the stratified cumulative models indicates the over-riding importance for the judgment of timeliness in government response for the UK-based sample both independently for each outcome and cumulatively. The uncertainty around whether or not participants or their family members had experienced SARS-CoV-2 infection similarly had a relationship to resilience and burnout (in this case, just the self) for the RoI-based subsample. This likely reflects differences in the way that the pandemic has evolved in each of these countries, and the subsequent impact this may have on frontline workers.

The present study provides a timely and important addition to the literature on the experiences of frontline workers during times of crisis. The study is set at a critical time during the pandemic in the UK and RoI, commencing data collection at pre-peak and continuing to post-peak during the first surge of a global pandemic. The sample size of the present study is also a strength, providing a robustness to the findings overall and by country subsample. The present study builds on existing literature to add to the overall picture of factors associated with heroic acts, providing personal and contextual understanding to various aspects of psychological health and wellbeing in a broad and atypical (for the literature) sample of frontline workers. To our knowledge, this is the first study to attempt to compare the experiences of frontline workers across countries, where there have been meaningful differences in pandemic strategy. Moreover, this is the first study to report on resilience, burnout, and wellbeing during a global pandemic in a broad and comprehensive conceptualization of frontline worker. Prior research into the SARS and H1N1 pandemics, which were comparatively less internationally devastating in both reach and depth of health and economic damage, have focused purely on healthcare workers, mostly those in hospital settings. Here, we not only incorporate healthcare workers in community and social care settings (such as care homes, and community healthcare hubs such as general practitioners), we also include other sectors of workers who have found themselves on the frontline: supermarket workers, teachers who have been supporting the children of keyworkers (in the UK), social workers, police officers, and testing station workers. The inclusion of these other workers into the consideration of their vulnerability to stress-related harm in their work is an important acknowledgment of the sacrifices they have made, and of their importance in supporting the population during such times.

There is a clear remit for resilient coping within the context of the welfare of frontline workers, thus a key recommendation from this work would be to focus on interventions that introduce or otherwise increase the utilization of such coping styles. The present findings give insight into the consequences of political strategy during such times and find that the lack of timeliness in the UK’s government response is also associated in the psychological welfare of its frontline workers. Whilst unpacking the direct and indirect influence of policy on behavior and health is difficult, these findings are consistent with research that shows relationships between policy decisions and health in other areas (Msetfi et al., 2018). The present work provides a theoretical contribution to the field also, by providing a greater understanding of the interplay between individual-level variables and contextual factors in relation to mental health. There are significant contributions to policy to be made from the present research. One clear indication is the need for governments to act in a timely way in response to such crises. The finding herein that the perception of timeliness of government response appears to be associated with poorer outcomes, specifically for those in the UK, provides a stark warning to UK-based organizations in frontline sectors that support is needed to protect these workers from burning out. The relatively slower response of the UK government to introducing effective measures to combat the spread of SARS-CoV-2 has had an apparent impact on infection rate, death toll, and now on the welfare of its most precious asset in such times: its frontline workers (Scally et al., 2020).

### Limitations and Future Research

The present study is limited by its cross-sectional perspective, and as such cannot determine causality with regard to the variables analyzed. As this present study is part of a longitudinal project, further work examining the long-term impact of the pandemic and these baseline factors will be determinable in future studies. Whilst the variables of interest were chosen in order to understand how they contribute to these mental wellbeing outcomes, any differences between the countries pre-pandemic in these variables cannot be accounted for this, however, will be addressed through through future longitudinal analyses to some extent. There are many other variables of importance and interest in the current pandemic in these workers — including levels of stress, locus of control, and more detailed assessments of attitudes toward government pandemic strategy — that were beyond the scope of the present study. Further, and more detailed, understanding of social support and the quality of frontline workers’ relationships with significant others will be of particular interest. The present research was conducted at a time when frontline workers were increasing their weekly working hours, and working in conditions that were increasingly demanding. It was, therefore, of ethical importance to ensure that we, the researchers, were able to derive meaningful answers in a time-effective manner.

There is also the issue of sample bias. The present sample, whilst sufficiently large and robust, is not demographically representative of the UK nor RoI, with respect to frontline worker profile or population. Moreover, the two subsamples are not even in size, providing a relative dominance of the UK-based sample in full-sample models. This has been partially addressed by assessing outcomes in country-based models but is nonetheless a limitation that has an impact on the interpretation of the findings. Further work with more diverse samples is warranted in any potential future crises. As part of a larger project, further analyses will be carried out on these and subsequent longitudinal data to explore sectoral and organizational level variables, as well as longer-term consequences of working on the frontline.

## Conclusion

The present study set out to understand what factors may be associated with the psychological welfare (as determined by resilience, burnout, and wellbeing) in a broad profile of frontline workers, beyond those in healthcare, during the Covid-19 pandemic. Further, we sought to understand whether government policy in dealing with the pandemic may have been associated with these outcomes by comparing frontline workers from the UK and RoI. To this end, we have found that the personal factors of presence of meaning in life, and resilient coping styles are associated with more positive welfare outcomes (i.e., higher resilience, lower burnout, and higher wellbeing), and search for meaning in life inversely associated. We also find that the perception of the timeliness of the government’s response to the pandemic appears to be an important factor in these outcomes in the UK-based sample. In stark contrast to the role that governments should be providing, in safeguarding and encouraging the resilience of all its citizens (Jetten et al., 2020), it appears that this has not been the case in the UK, but may well be in the RoI, if at least during the period of time assessed through the present study. Situated in the context of the proportionally higher morbidity and mortality rate that the UK has experienced during the pandemic, the present findings suggest that the welfare, and lower overall wellbeing, of UK frontline workers may also be part of this fallout. These findings offer insights into the correlates of wellbeing, burnout and resilience of frontline workers during the Covid-19 pandemic during the acute phase. This information can be used to plan for future waves of Covid-19 and inevitable future societal disasters where we will again rely on heroic efforts of workers to keep our societies afloat.

## Data Availability Statement

The datasets generated for this study are not readily available because the data will be hosted online at OSF after the completion of all publications from the project. Requests to access the datasets should be directed to RS, rsumner@glos.ac.uk.

## Ethics Statement

The studies involving human participants were reviewed and approved by the Natural & Social Sciences Research Ethics Panel at the University of Gloucestershire. The patients/participants provided their written informed consent to participate in this study.

## Author Contributions

RS and EK were responsible for the development and implementation of the study protocol, the analysis, and final writing of this report. Both authors contributed to the article and approved the submitted version.

## Dedication

The COVID-19 pandemic has taken many lives, and will continue to do so. Among those are the lives of frontline workers – named and unnamed – who, for a variety of reasons, unfortunately felt that they had no choice but to take their own lives. This work is dedicated to their memory, to the incredible sacrifices and efforts made by all frontline workers worldwide, and to all those lives lost in the pandemic.

## Conflict of Interest

The authors declare that the research was conducted in the absence of any commercial or financial relationships that could be construed as a potential conflict of interest.

## Footnotes

1 onlinesurveys.ac.uk

2 www.cv19heroes.com

3 https://osf.io/hcr6x

4 Comparisons across sectors are due to be conducted in future analyses of these data as part of the larger project.
