# Supplementary material for: Grace Under Pressure: Resilience, Burnout, and Wellbeing in Frontline Workers in the United Kingdom and Republic of Ireland During the SARS-CoV-2 Pandemic
Source: Front Psychol. 2021 Jan 27;11:576229. doi: 10.3389/fpsyg.2020.576229 (PMC7874970; doi:10.3389/fpsyg.2020.576229)
Supplement: Supplementary file 1 [file Table_1.docx]

Table S1. Regression models examining separate contributions of personal and pandemic factors for resilience, burnout, and wellbeing in the whole frontline worker sample.

|  |  | Model S1 | | | | | Model S2 | | | | | Model S3 | | | | |
| --- | --- | --- | --- | --- | --- | --- | --- | --- | --- | --- | --- | --- | --- | --- | --- | --- |
|  |  | Resilience | | | | | Burnout | | | | | Wellbeing | | | | |
|  |  | *F*_(13, 1207)_=33.37, *p*<0.001, *R^2^*=.26, *R^2^*_adj_=.26 | | | | | *F*_(14, 1206)_=21.01, *p*<0.001, *R^2^*=.20, *R^2^*_adj_ =.19 | | | | | *F*_(15, 1205)_=84.85, *p*<0.001, *R^2^*=.51, *R^2^*_adj_ =.51 | | | | |
|  |  | β | *t* | *p* | 95% CI | | β | *t* | *p* | 95% CI | | β | *t* | *p* | 95% CI | |
|  |  |  |  |  | Lower | Upper |  |  |  | Lower | Upper |  |  |  | Lower | Upper |
|  | Partnership status | 0.002 | 0.037 | 0.971 | -0.087 | 0.090 | 1.494 | 2.669 | **0.008** | 0.396 | 2.592 | 0.166 | 0.754 | 0.451 | -0.266 | 0.597 |
|  | Caring status | -0.018 | -0.409 | 0.683 | -0.103 | 0.067 | 0.865 | 1.61 | 0.108 | -0.189 | 1.919 | -0.164 | -0.778 | 0.437 | -0.577 | 0.249 |
|  | MLQ Presence | 0.024 | 6.641 | **<0.001** | 0.017 | 0.031 | -0.299 | -6.558 | **<0.001** | -0.388 | -0.209 | 0.150 | 8.279 | **<0.001** | 0.115 | 0.186 |
|  | MLQ Search | -0.019 | -6.767 | **<0.001** | -0.024 | -0.013 | 0.155 | 4.414 | **<0.001** | 0.086 | 0.224 | -0.008 | -0.549 | 0.583 | -0.035 | 0.020 |
|  | Altruism | -0.003 | -1.379 | 0.168 | -0.008 | 0.001 | 0.033 | 0.093 | 0.926 | -0.054 | 0.059 | 0.022 | 1.927 | 0.054 | 0.000 | 0.044 |
|  | Resilient Coping | 0.111 | 14.022 | **<0.001** | 0.096 | 0.127 | 0.137 | 1.293 | 0.196 | -0.071 | 0.346 | 0.265 | 6.354 | **<0.001** | 0.183 | 0.346 |
|  | Resilience* |  |  |  |  |  | -2.738 | -7.668 | **<0.001** | -3.438 | -2.037 | 1.565 | 10.933 | **<0.001** | 1.284 | 1.846 |
|  | Burnout** |  |  |  |  |  |  |  |  |  |  | -0.182 | -16.117 | **<0.001** | -0.204 | -0.160 |
| Government response rating | Appropriate | -0.008 | -0.725 | 0.469 | -0.030 | 0.014 | 0.024 | 0.175 | 0.861 | -0.25 | 0.299 | -0.052 | -0.942 | 0.346 | -0.159 | 0.056 |
|  | Timely | 0.014 | 1.188 | 0.235 | -0.009 | 0.036 | -0.335 | -2.357 | **0.019** | -0.613 | -0.056 | 0.159 | 2.856 | **0.004** | 0.050 | 0.269 |
|  | Effective | 0.007 | 0.531 | 0.596 | -0.018 | 0.032 | 0.061 | 0.389 | 0.697 | -0.248 | 0.371 | 0.051 | 0.827 | 0.408 | -0.070 | 0.172 |
| CV19 Infection certainty | Self | -0.079 | -2.293 | **0.022** | -0.147 | -0.011 | 1.148 | 2.676 | **0.008** | 0.306 | 1.990 | -0.006 | -0.038 | 0.970 | -0.337 | 0.324 |
|  | Family | 0.046 | 1.438 | 0.151 | -0.017 | 0.108 | -0.324 | -0.819 | 0.413 | -1.098 | 0.451 | -0.021 | -0.135 | 0.892 | -0.324 | 0.283 |
|  | Friends | -0.002 | -0.092 | 0.926 | -0.052 | 0.048 | 0.753 | 2.387 | **0.017** | 0.134 | 1.373 | -0.031 | -0.248 | 0.804 | -0.274 | 0.212 |
|  | Co-Workers | 0.012 | 0.428 | 0.669 | -0.042 | 0.066 | 0.800 | 2.343 | **0.019** | 0.130 | 1.469 | 0.207 | -1.547 | 0.122 | -0.470 | 0.056 |
|  |  |  |  |  |  |  |  |  |  |  |  |  |  |  |  |  |
|  | *Models 2 and 3 only | |  |  |  |  |  |  |  |  |  |  |  |  |  |  |
|  | **Model 3 only | |  |  |  |  |  |  |  |  |  |  |  |  |  |  |

Significant differences are highlighted **in bold**.

^†^Burnout models were fit using the total (unmeaned) Bergen Burnout Inventory score.

MLQ=Meaning in Life Questionnaire

Resilient coping refers to specific adaptational styles associated with coping that are supportive of resilience. Resilience refers to the status of having successfully handled stressful situations.

Table S2. Stratified regression models to examine the combined associations of personal and pandemic factors for the United Kingdom (UK)-based (a) and Republic of Ireland (RoI)-based (b) subsamples for resilience (S4), burnout (S5), and wellbeing (S6)

|  |  | Model S4a - UK | | | | | Model S5a - UK | | | | | Model S6a - UK | | | | |
| --- | --- | --- | --- | --- | --- | --- | --- | --- | --- | --- | --- | --- | --- | --- | --- | --- |
|  |  | Resilience | | | | | Burnout | | | | | Wellbeing | | | | |
|  |  | *F*_(13, 809)_=23.31, *p*<0.001, *R^2^*=.273, *R^2^*_adj_=.261 | | | | | *F*_(14, 808)_=11.87, *p*<0.001, *R^2^*=.17, *R^2^*_adj_ =.16 | | | | | *F*_(15, 807)_=55.24, *p*<0.001, *R^2^*=.51, *R^2^*_adj_ =.50 | | | | |
|  |  | β | *t* | *p* | 95% CI | | β | *t* | *p* | 95% CI | | β | *t* | *p* | 95% CI | |
|  |  |  |  |  | Lower | Upper |  |  |  | Lower | Upper |  |  |  | Lower | Upper |
|  | Partnership status | -0.010 | -0.189 | 0.850 | -0.118 | 0.097 | 1.470 | 2.180 | **0.030** | 0.146 | 2.795 | 0.034 | 0.128 | 0.898 | -0.496 | 0.565 |
|  | Caring binary | 0.016 | 0.299 | 0.765 | -0.087 | 0.118 | 0.718 | 1.119 | 0.264 | -0.542 | 1.979 | -0.241 | -0.941 | 0.347 | -0.745 | 0.262 |
|  | MLQ Presence | 0.022 | 5.119 | **<0.001** | 0.014 | 0.030 | -0.270 | -5.032 | **<0.001** | -0.375 | -0.164 | 0.163 | 7.516 | **<0.001** | 0.121 | 0.206 |
|  | MLQ Search | -0.021 | -6.064 | **<0.001** | -0.027 | -0.014 | 0.175 | 4.105 | **<0.001** | 0.091 | 0.258 | -0.010 | -0.557 | 0.577 | -0.043 | 0.024 |
|  | Altruism | -0.005 | -1.667 | 0.096 | -0.010 | 0.001 | -0.024 | -0.725 | 0.469 | -0.091 | 0.042 | 0.024 | 1.764 | 0.078 | -0.003 | 0.050 |
|  | Resilient Coping | 0.113 | 12.093 | **<0.001** | 0.095 | 0.132 | 0.247 | 1.974 | **0.049** | 0.001 | 0.493 | 0.239 | 4.771 | **<0.001** | 0.141 | 0.337 |
|  | Resilience* |  |  |  |  |  | -2.403 | -5.553 | **<0.001** | -3.252 | -1.553 | 1.658 | 9.422 | **<0.001** | 1.312 | 2.003 |
|  | Burnout** |  |  |  |  |  |  |  |  |  |  | -0.182 | -12.975 | **<0.001** | -0.21 | -0.155 |
| Government response rating | Appropriate | -0.018 | -1.204 | 0.229 | 0.002 | 0.062 | 0.107 | 0.594 | 0.552 | -0.246 | 0.46 | -0.071 | -0.985 | 0.325 | -0.212 | 0.070 |
|  | Timely | 0.032 | 2.069 | **0.039** | -0.021 | 0.042 | -0.529 | -2.779 | **0.006** | -0.903 | -0.155 | 0.157 | 2.056 | **0.040** | 0.007 | 0.307 |
|  | Effective | 0.010 | 0.629 | 0.529 | -0.152 | 0.008 | -0.001 | -0.004 | 0.997 | -0.389 | 0.387 | 0.066 | 0.840 | 0.401 | -0.089 | 0.221 |
| CV19 Infection certainty | Self | -0.072 | -1.773 | 0.077 | -0.054 | 0.092 | 0.572 | 1.144 | 0.253 | -0.410 | 1.554 | 0.004 | 0.020 | 0.984 | -0.388 | 0.396 |
|  | Family | 0.019 | 0.511 | 0.610 | -0.048 | 0.075 | 0.167 | 0.364 | 0.716 | -0.733 | 1.066 | 0.245 | 1.339 | 0.181 | -0.114 | 0.604 |
|  | Friends | 0.014 | 0.430 | 0.668 | -0.042 | 0.096 | 0.455 | 1.173 | 0.241 | -0.306 | 1.215 | -0.233 | -1.507 | 0.132 | -0.537 | 0.071 |
|  | Co-Workers | 0.027 | 0.769 | 0.442 | -0.656 | 1.866 | 0.532 | 1.235 | 0.217 | -0.313 | 1.215 | -0.108 | -0.626 | 0.531 | -0.445 | 0.230 |
|  |  |  |  |  |  |  |  |  |  |  |  |  |  |  |  |  |
|  |  |  |  |  |  |  |  |  |  |  |  |  |  |  |  |  |
|  |  | Model S4b - RoI | | | | | Model S4b - RoI | | | | | Model S6b - RoI | | | | |
|  |  | Resilience | | | | | Burnout | | | | | Wellbeing | | | | |
|  |  | *F*_(13, 384)_=10.70, *p*<0.001, *R^2^*=.27, *R^2^*_adj_=.24 | | | | | *F*_(14, 383)_=11.82, *p*<0.001, *R^2^*=.30, *R^2^*_adj_ =.28 | | | | | *F*_(15, 382)_=29.07, *p*<0.001, *R^2^*=.53, *R^2^*_adj_ =.52 | | | | |
|  |  | β | *t* | *p* | 95% CI | | β | *t* | *p* | 95% CI | | β | *t* | *p* | 95% CI | |
|  |  |  |  |  | Lower | Upper |  |  |  | Lower | Upper |  |  |  | Lower | Upper |
|  | Partnership status | 0.040 | 0.490 | 0.624 | -0.119 | 0.199 | 1.702 | 1.711 | 0.088 | -0.254 | 3.659 | 0.641 | 1.696 | 0.091 | -0.102 | 1.383 |
|  | Caring binary | -0.107 | -1.341 | 0.181 | -0.264 | 0.050 | 0.902 | 0.917 | 0.360 | -1.032 | 2.836 | -0.262 | -0.703 | 0.483 | -0.994 | 0.471 |
|  | MLQ Presence | 0.028 | 4.154 | **<0.001** | 0.015 | 0.042 | -0.376 | -4.363 | **<0.001** | -0.546 | -0.207 | 0.096 | 2.862 | **0.004** | 0.030 | 0.161 |
|  | MLQ Search | -0.015 | -3.026 | 0.003 | -0.025 | -0.005 | 0.116 | 1.843 | 0.066 | -0.008 | 0.239 | -0.01 | -0.429 | 0.668 | -0.057 | 0.037 |
|  | Altruism | 0.005 | 0.403 | 0.687 | -0.007 | 0.011 | 0.072 | 1.289 | 0.198 | -0.038 | 0.181 | 0.015 | 0.711 | 0.478 | -0.026 | 0.056 |
|  | Resilient Coping | -0.009 | 7.002 | **<0.001** | 0.077 | 0.137 | -0.220 | -1.103 | 0.271 | -0.613 | 0.172 | 0.309 | 4.082 | **<0.001** | 0.160 | 0.457 |
|  | Resilience* |  |  |  |  |  | -3.171 | -5.050 | **<0.001** | -4.405 | -1.936 | 1.375 | 5.605 | **<0.001** | 0.892 | 1.857 |
|  | Burnout** |  |  |  |  |  |  |  |  |  |  | -0.192 | -9.913 | **<0.001** | -0.230 | -0.154 |
| Government | Appropriate | 0.001 | 0.253 | 0.800 | -0.031 | 0.040 | -0.145 | -0.644 | 0.520 | -0.586 | 0.297 | -0.051 | -0.596 | 0.552 | -0.218 | 0.116 |
|  | Timely | -0.119 | -0.492 | 0.623 | -0.046 | 0.028 | -0.251 | -1.085 | 0.278 | -0.707 | 0.204 | 0.067 | 0.761 | 0.447 | -0.106 | 0.239 |
|  | Effective | 0.112 | 0.053 | 0.958 | -0.041 | 0.043 | 0.234 | 0.894 | 0.372 | -0.281 | 0.750 | 0.061 | 0.619 | 0.536 | -0.134 | 0.257 |
| CV19 Infection certainty | Self | -0.119 | -1.769 | 0.078 | -0.252 | 0.013 | 2.765 | 3.324 | **0.001** | 1.129 | 4.401 | 0.013 | 0.041 | 0.967 | -0.615 | 0.641 |
|  | Family | 0.112 | 1.669 | 0.096 | -0.020 | 0.244 | -0.672 | -0.811 | 0.418 | -2.303 | 0.958 | -0.610 | -1.942 | 0.053 | -1.227 | 0.008 |
|  | Friends | -0.033 | -0.751 | 0.453 | -0.119 | 0.053 | 1.263 | 2.345 | **0.020** | 0.204 | 2.322 | 0.413 | 2.011 | **0.045** | -0.009 | 0.816 |
|  | Co-Workers | -0.033 | -0.075 | 0.940 | -0.094 | 0.087 | 1.206 | 2.123 | **0.034** | 0.089 | 2.322 | -0.474 | -2.192 | **0.029** | -0.898 | -0.049 |
|  |  |  |  |  |  |  |  |  |  |  |  |  |  |  |  |  |
|  | *Models S5 and S6 only | |  |  |  |  |  |  |  |  |  |  |  |  |  |  |
|  | **Model S6 only | |  |  |  |  |  |  |  |  |  |  |  |  |  |  |

Significant differences are highlighted **in bold**.

^†^Burnout models were fit using the total (unmeaned) Bergen Burnout Inventory score.

MLQ=Meaning in Life Questionnaire

Resilient coping refers to specific adaptational styles associated with coping that are supportive of resilience. Resilience refers to the status of having successfully handled stressful situations.
